# Implementation of ‘matrix support’ (collaborative care) to reduce asthma and COPD referrals and improve primary care management in Brazil: a pilot observational study

**DOI:** 10.1038/npjpcrm.2016.47

**Published:** 2016-08-18

**Authors:** Sonia Maria Martins, William Salibe-Filho, Luís Paulo Tonioli, Luís Eduardo Pfingesten, Patrícia Dias Braz, Juliet McDonnell, Siân Williams, Débora do Carmo, Jaime Correia de Sousa, Hilary Pinnock, Rafael Stelmach

**Affiliations:** 1 Department of Community Health of the Faculty of Medicine of ABC (FMABC), São Bernardo do Campo, São Paulo, Brazil; 2 Respiratory Group Brazilian Society of Family and Community Medicine (SBMFC), São Bernardo do Campo, São Paulo, Brazil; 3 Pulmonology Service of the University São Camilo Medical School, São Paulo, Brazil; 4 PHC Division-Health Secretary, São Bernardo do Campo, São Paulo, Brazil; 5 International Primary Care Respiratory Group (IPCRG), Westhill, Scotland, UK; 6 Specialized Department, São Bernardo do Campo, São Paulo, Brazil; 7 Community Health, Life and Health Sciences Research Institute (ICVS), School of Health Sciences, University of Minho, Portugal, Horizonte Family Health Unit, Matosinhos, Portugal; 8 Allergy and Respiratory Research Group, Usher Institute of Population Health Sciences and Informatics, University of Edinburgh, Edinburgh, UK; 9 Pulmonary Division-Heart Institute (InCor)—Hospital da Clinicas da Faculdade de Medicina da Universidade de São Paulo, Sao Paulo, Brazil

## Abstract

Asthma and chronic obstructive pulmonary disease (COPD) are leading causes of hospitalisation and death in the city of Sao Bernardo do Campo. The municipality had difficulties in sustaining a pulmonology specialist team. Local policy has strengthened the knowledge of the primary care teams to improve the management of these diseases. Our aim is to pilot the implementation of an educational intervention based on collaborative care focused on reducing respiratory-related referrals. We implemented ‘matrix support’: a Brazilian collaborative educational intervention promoting specialist training and support for primary care physicians in three health territories with the highest number of referrals. Clinicians and nurses from primary care attended an 8-h workshop. The backlog of respiratory referrals was prioritised, where Asthma and COPD represented 70% of referral reasons. Initially, pulmonologists held joint consultations with physicians and nurses; as confidence grew, these were replaced by round-table note-based case discussions. The primary outcome was the number of asthma and COPD referrals. Almost all primary healthcare professionals in the three areas (132 of 157–87%) were trained; 360 patients were discussed, including 220 joint consultations. The number of respiratory referrals dropped from 290 (the year before matrix support) to 134 (the year after) (*P*<0.05). Referrals for asthma/COPD decreased from 13.4 to 5.4 cases per month (*P*=0.09) and for other lung diseases from 10.8 to 5.3 cases per month (*P*<0.05). Knowledge scores showed a significant improvement (*P*<0.001). Matrix-support collaborative care was well-accepted by primary care professionals associated with improved knowledge and reduced respiratory referrals. The initiative attracted specialists to the region overcoming historical recruitment problems.

## Introduction

To cope with the epidemic of non-communicable diseases,^1^ health policy in many countries is promoting ambulatory care models that reduce reliance on hospitals by building a sustainable network of community-based primary care support.^2^ Recent studies in Brazil have demonstrated that the prevalence of asthma among adolescents is 12.4%^3^ and the prevalence of chronic obstructive pulmonary disease (COPD) in people over 40 years is 15.8%,^4^ 70% of whom were undiagnosed.^5^ As part of Brazilian government policy to address the growth in non-communicable diseases, including chronic respiratory diseases, primary care has been strengthened through a Family Health Strategy (FHS).^2^ FHS teams with a family physician, nurse, nursing assistant and community health workers provide holistic care to families with a focus on health promotion, disease prevention, as well as diagnosis, treatment and rehabilitation. A key strategy has been to strengthen family medicine’s academic credentials and thus the credibility of generalism as a medical speciality.^6^

Brazil has a public health service that provides free access to healthcare for almost 70% of the population, managed at the municipality level. A private complementary health structure is used by the other 30%, based on insurance contracts. The public system is based in Basic Health Units (BHU), within which FHS teams are located.

This paper describes a pilot implementation project in São Bernardo do Campo city, a municipality in the state of São Paulo-Brazil, which aimed to maximise the resources available for asthma and COPD care by using a model known as ‘matrix support’. Matrix support is a model of collaborative care that originated in Brazilian Mental Health settings.^7–9^ It promotes shared care, improving communication between primary care and specialised care to support patient-centred care in the community. This model of collaborative care builds on the principles of academic detailing and educational outreach, which have been shown to be effective in promoting improved care,^10,11^ by facilitating collaboration to support a secondary to primary shift in care. Specific clinical aims include increasing recognition and diagnosis of health problems and improving the appropriateness of referrals to specialist care.^12^ Effective management in primary care has been shown to reduce the likelihood of hospitalisation in asthma and COPD in Brazil,^13^ and in other healthcare systems (for example, in the Finnish asthma programme).^14^

For a period of 2 years (2011–12), the municipality where the study was applied had difficulties in recruiting and sustaining a pulmonology specialist network.^15^ The absence of adequate medical infrastructure retaining clinicians where they are most needed, low professional salaries and poor integration/interaction between levels of care resulting in a plethora of referral cases to specialists—most of them with mild diseases—are among the multifactorial causes.

The aim of this study was to implement an educational intervention based on the concept of matrix support and to evaluate its impact on the management of asthma and COPD in São Bernardo do Campo’s primary care health setting. The principal objective was to improve knowledge and collaboration among primary healthcare workers, leading to the reduction and improvements in the quality of referrals.

## Results

This was an evaluation of the routine implementation of a model of care, and ethics approval was not required. The programme started in April 2013. Local BHU meetings were conducted from May 2013 to April 2014.

### Process of implementation

A total of 132 health professionals (87% of the 157 primary care professionals in the 3 territories) were trained at the workshops, the majority of whom were general physicians, some family physicians and nurses. (Table 1) Of the 56 physicians who attended the workshop, 45 took part in the joint consultations, and 36 attended a case discussion.

**Figure 1:**
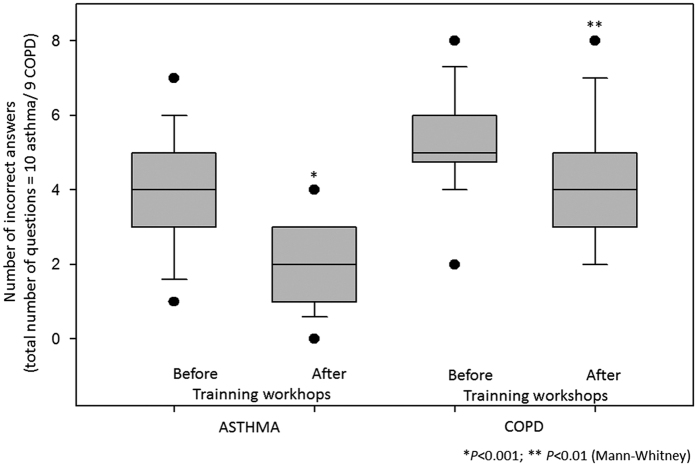
Number of incorrect answers on the knowledge questionnaire comparing before and after training workshop in both asthma and COPD.

The knowledge questionnaires were applied before and immediately after the training seminars, and completed by 88% of the workshop attendees. The results showed greater prior knowledge of asthma compared with COPD, but both increased after training. The professionals’ level of knowledge before intervention in the three territories showed a similar pattern. The number of errors decreased significantly both for asthma and COPD questions (Figure 1). Before intervention, up to 90% of the answers to the open question ‘do you feel secure to diagnose and treat COPD—yes/no, why?’ was no. The four main reasons were as follows: (a) there is no local training; (b) I have little knowledge about COPD; (c) it is very complex because most of the time there are co-morbidities; (d) in most cases the disease does not stabilise even with medications.

A total of 360 patients attended, including 220 joint consultations, which is a higher number than the original referral backlog because new patients were identified by the teams during the course of the programme. After 3 months of the programme, each physician had participated on average in two joint consultations. Professionals felt more knowledgeable and confident to manage asthma and COPD patients alone, and bringing new cases about other patients with diagnostic or therapy uncertainty for clinical discussions at 50 round-table discussions. During these discussions, some professionals highlighted the joint consultations as the ‘best part’ of the matrix support.

### Impact of the intervention

**Figure 2:**
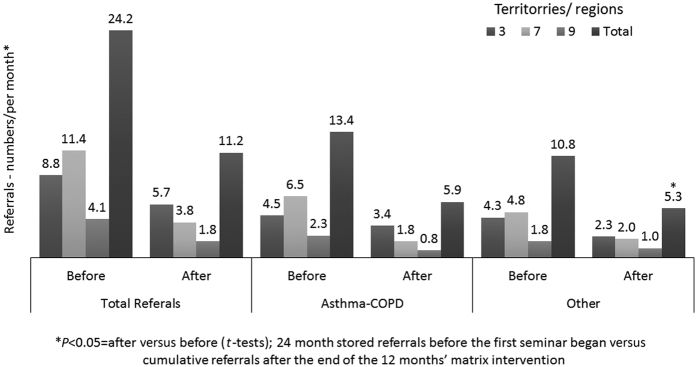
Before and after referrals (*n* per month)—by territory—before and after matrix support (24 months versus 12 months).

The number of referrals to secondary pulmonology care dropped significantly for asthma, COPD and other lung diseases (581 over the previous 24 months to 134 in the 12 months after the workshops, *P*<0.05). Over the same period, the rate of referral for asthma and COPD decreased from 13.4 to 5.4 cases per month and for other lung diseases from 10.8 to 5.3 per month (*P*<0.05) (Figure 2).

The dispensing of beclomethasone propionate inhaler canisters (MDI 250 mcg or DPI 50 mcg) from the three territories increased by 43% (from 804 canisters to 1,150) in the 5 months after the matrix programme started, which is an indirect sign that patients with persistent asthma had not been properly treated previously.

**Figure 3:**
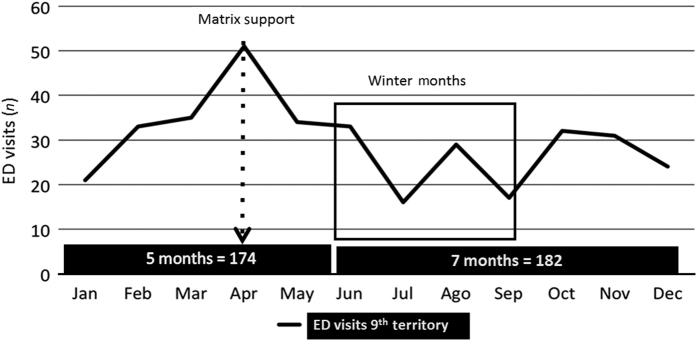
Emergency Department Visits: before (5 months) and during (7 months)—Territory 9.

The rate of emergency department attendances in the 7 months after the workshop (*n*=182; 26 per month) was not significantly different from the 5 months before the workshop (*n*=174; 35 per month), Figure 3. In this territory, the emergency department is the only one available.

### Unplanned consequences

Before the matrix-support programme, there was only one pulmonologist specialising in the management of tuberculosis. The project attracted pulmonologists to the county, and at the end of the programme four pulmonologists were contributing to ambulatory care and matrix support.

The matrix educational process in respiratory diseases was associated with an increased demand for pulmonary function tests by primary care teams. Requests for spirometry increased by 27.5% (from 1,200 to 1,530 per year), and extra capacity had to be provided.

Before the programme, none of the BHUs offered accredited smoking cessation support. After the programme, 10 smoking cessation support units were available, with another 11 in the process of training and accreditation.

## Discussion

### Main findings

Implementation of matrix-supported collaborative care engaged 87% of primary care clinicians and nurses in training and mentorship, and was perceived to have increased their confidence in managing common respiratory conditions. There was a reduction in referrals for specialist opinion associated with the launch of the initiative. An unplanned benefit was that the programme attracted pulmonologists to jobs in the previously under-served county, potentially increasing the sustainability of the programme.

### Interpretation of findings in relation to previously published work

Matrix support was adapted successfully from its origins in mental healthcare to asthma and COPD management. Gonçalves *et al*.,^12^ in a multicentre study, showed no overall impact from a shared care initiative to improve recognition of mental health problems. Onocko-Campos *et al*.,^16^ in a cluster analysis of the outcomes of health promotion and mental health outcomes in primary care units in South Eastern Brazil, identified positive advances in units with higher implementation of innovative community integration strategies. Subsequent qualitative work confirmed the challenges of overcoming the barriers to communication, both between levels of care and within the teams. Highlighted by the President of the World Organization of Family Doctors as building effective partnerships,^17^ successful units implemented aspects of matrix support (regular meetings, participation of the entire team and collaboration between specialised and primary care professionals).

A literature review using the term ‘matrix support’ did not find studies outside Brazil. The pivotal concept of collaborative and integrated care management in asthma and COPD, however, is widely represented in the international literature with examples in the respiratory field from Australia,^18^ Canada,^19,20^ Finland,^14^ Denmark,^21^ the UK^22^ and US.^23^ These initiatives encompass integrated networks of patient-centred medical homes^23^ and physician–pharmacist collaboration, which improved outcomes in people with asthma.^24^ For COPD patients, collaborative healthcare is often related to promoting patient behavioural change and supporting self-management.^19,20^ An Australian primary care collaboration reduced hospitalisations,^18^ and a multidisciplinary community-based group in the UK increased referrals to pulmonary rehabilitation and reduced hospital admissions in a deprived ethnically diverse area.^22^ Integrated care underpins a multi-sectorial initiative for respiratory disease in Europe.^25^

Our study illustrates the importance of undertaking and publishing implementation studies. Initiatives on academic detailing or educational outreach carried out in developed countries^10,11,26^ may not relate to the health service context in low- and middle-income countries. Implementation in countries (such as Brazil) where family medicine is an emerging speciality has specific challenges, as established hierarchies are challenged^27^ and the boundaries shift between new professional roles. The WHO’s Package of Essential Non-Communicable Disease Interventions^28^ advocates collaborative integrated care to improve case detection, diagnosis and management of respiratory disease, but there are a few implementation studies in low- and middle-income countries testing educational strategies to improve capacity and quality of care. An important exception is an interactive, case-based, trainer on-site service designed to implement improved care for respiratory diseases in South Africa, which demonstrates the substantial impact of professional training that conforms to modern adult learning methods.^29,30^

In the Brazilian context of a new national health system - in which the number of physicians per 1,000 population is lower than most high-income countries,^31^ respect for Family Medicine is still evolving,^6^ and Primary Healthcare strategies are still learning how to substitute for the traditional specialised care - the link between levels of care is truly fragile. An important part of the reorganisation is knowledge transfer, and promotion of mutual collaboration, Matrix support not only adopts the evidence-based strategy of educational outreach^11^ or academic detailing,^10,26^ but used shared consultations to further our core aim of shifting collaboratively the boundary of care between secondary and primary care. Our approach may have resonance for other healthcare systems in which primary care is striving to establish a role.^6^

Reflecting the pivotal importance of collaboration with specialist colleagues, the participants in the matrix-support initiative identified the joint consultations as the ‘best component of the intervention’. While learning was the key aim of this joint working, it may also have served to break down traditional hierarchical structures recognised in other contexts as a barrier to primary healthcare.^6^

### Strengths and limitations of this study

Building on international evidence of the benefits of collaborative care, this study tailored a strategy designed for Brazilian primary healthcare to address a particular local challenge. The project was perceived as having had an impact on the empowerment of primary healthcare professionals in dealing more autonomously with chronic respiratory diseases, potentially improving the efficiency of the management of respiratory disease.

There were some methodological limitations in this real-life study.^32^ Some outcomes could not be measured in all BHU or in all the territories, and some end points (such as hospitalisations and mortality) were only available at the city level and could not therefore be included. Our primary end point (referrals) was a concrete and easy-to-measure outcome within the healthcare system, but further work is needed to examine the quality of the diagnoses made by the trained primary care clinicians in the patients they did not refer and the sustainability of change. The lack of control territories means that we cannot deduce causality. We acknowledge that, compared with physicians, nurses and other non-medical health workers did not participate as actively in this collaborative care programme possibly because they were not given time to attend/participate.

Participation was strongly promoted at the municipality level, and only 13% of primary care physicians did not attend the workshop. In the context of implementation studies, fidelity and adaptation are both important. There will have been some variation in the delivery of the workshops and the focus of joint consultations; however, we ensured that trainers were trained and familiar with Brazilian guidelines^33^ and provided mentorship to support the primary care clinicians as they implemented the learning within their clinical practice.

Although the professional knowledge questionnaire has not formally been validated, the questions were based on questionnaires that were widely used in Brazilian medical education.

### Implications for future research, policy and practice

Our findings support evidence that achieving change in models of healthcare requires a collaborative approach that not only provides training in new skills but seeks to enhance and support the role of professionals within the healthcare system, while raising awareness of the importance of a condition. Some lessons learnt from our experience of implementing matrix support in the context of respiratory care include the following:

- The insecurity and perceived inability of professionals in primary care to manage asthma and COPD can be addressed by the collaborative approach of matrix support (especially joint consulting), which promoted trusting relationships between professionals and was associated with a reduction in referrals for specialist care.
- Our intervention was more successful at engaging doctors than other healthcare professionals; future projects will need to invest more in promoting multidisciplinary care, empowering nurses and other non-physician professionals in PHC as a ‘front line’ of care in all chronic diseases.
- Further work is needed to examine the quality of the diagnoses made by the trained primary care clinicians in the patients they did not refer, and the sustainability of the change.

Sustainability needs to be considered in future projects: strengthening the involvement of nurses and allied healthcare professionals is an option to ensure the care and continuity of care because they form the bulk of the workforce in health, and the turnover in employment is less than in physicians.

### Conclusion

Matrix-support collaborative care, as adapted and implemented in this study, was perceived as an effective educational tool both for improving knowledge of asthma and COPD and for promoting a significant change in the relationship between primary and secondary care. The associated decrease in the number of referrals for pulmonology specialist advice suggests more confidence and sense of importance in primary care management. Healthcare systems faced with the challenge of shifting the care of people with long-term conditions from established specialist services to an emerging primary care should consider implementing collaborative educational strategies.

## Materials and methods

### Context for the intervention

São Bernardo do Campo city has almost 800,000 inhabitants, with a Human Development Index of 0.86, higher than the average of 0.74 of Brazil.^34^ The Human Development Index is a summary measure of average achievement in life expectancy, educational standards and standard of living; an HDI of 0.76 puts Brazil in the category of ‘high human development’ (as opposed to very high, medium, low categories).^35^ Just under half (45%) of the population is covered by the FHS.^34^ The city is divided into nine health territories, with a public health network considered adequate for the needs of the population, spending R$ 1,000 (300 US$ equivalent) per inhabitant per year to cover all healthcare costs.^34^ Local and national health data show that asthma and COPD (ICD X—J45&J44) are the combined second leading causes of hospitalisation and the third leading causes of death.^36^

Almost 30% of Brazilian physicians work in the State of São Paulo, giving coverage of 2.64 per 1,000 per inhabitants, but only half of them work in primary care as general physicians or family physicians (the latter are vocationally trained in the speciality of family medicine). Fewer than 2% of physicians in Sao Paulo State were respiratory specialists.^37^ An excessive number of respiratory-related referrals from primary care (70% of the 1,104 referrals were for asthma or COPD) has further compounded the challenge to healthcare services.^15^ The intervention described in this paper was developed and promoted at the municipal level to address this challenge by creating a local programme to support chronic respiratory disease diagnosis and care management.

### Local healthcare organisation

Three of the nine health territories in São Bernardo do Campo were chosen to receive the multidisciplinary educational intervention based on ‘matrix support’. Selection was based on the number of referrals to pulmonology. Over the 2 years before the intervention, half the referrals in the city were from these territories and most of the referrals (almost 70%)^15^ were related to asthma and/or COPD. These 3 territories have 11 BHU and serve ~231,000 people. Other reasons for choosing these areas were the long distance/difficult access (45 min per 20 km) to the downtown location of the city outpatient department. In addition, part of the ninth territory is located in a socially vulnerable rural area; during the design of the study, a woman from one of the territories died because of asthma, fuelling population demand for improvements.

### Implementation of the respiratory matrix collaborative intervention

**Figure 4:**
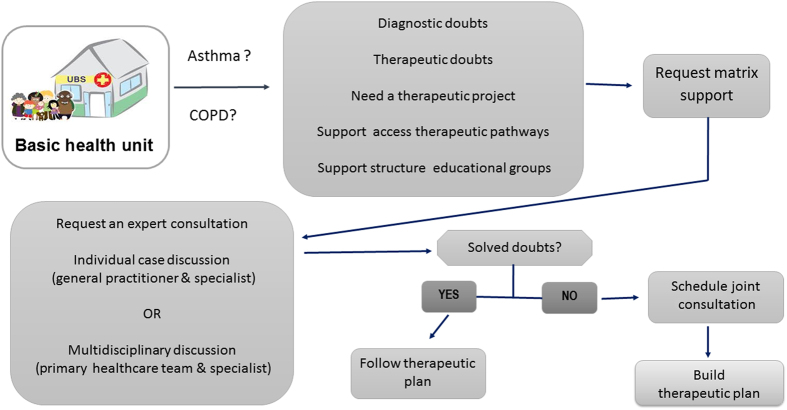
Flow chart of support matrix in asthma and COPD.

The structure of the respiratory matrix collaborative care model was based on two previous mental health studies,^12,16^ and is summarised in Figure 4. Implementation proceeded in three stages:

1. First, the primary care teams in partnership with pulmonologists prioritised the backlog of 581 referrals. Criteria for priority scheduling were the severity of the cases based on referral information, and how long they had already been waiting. The central regulation office returned all the referrals of patients waiting for pulmonology appointments to each BHU. Telephone contact was made with all the patients, advising them about the re-scheduling of the consultations, and community health workers confirmed this on home visits. This process was designed to be collaborative and to involve the network of professionals. For example, the understanding and commitment of the BHU manager, both to the matrix initiative and regarding the importance of primary care management of asthma and COPD, was essential for ensuring successful execution of this process locally.
2. The second stage was a series of themed mandated management workshops each lasting 8 h for family doctors, nurses and managers (Table 2). The purpose of the workshops was to raise professional awareness of the importance of asthma and COPD management within the context of public health and primary healthcare, and it included clinical case discussion. The standardised workshops were carried out in 11 BHUs in the 3 territories. The presentations and discussions were led by three pulmonologists and one primary care physician expert on matrix support and based on international asthma and COPD guidelines^38,39^ and on the Brazilian Ministry of Health Official Book ‘Chronic Respiratory Disease (asthma, COPD, rhinitis) for primary care'.^33^ Leaders attended previous 'train the trainers' meetings to guarantee fidelity between them. During the training sections, we reinforced the need to ‘build a network of responsible professionals’^14^ specifically by strengthening the role of nurses and collaborating across traditional boundaries to implement effective and efficient protocols for accessing pulmonology care.
3. The third stage consisted of a practical approach for primary care physicians through mentorship from pulmonologists. Specifically, this involved joint consultations for patients’ first visits, followed by individual case discussions and/or round-table note-based case discussions without patients. Agendas for the joint consultations were organised after contact with the patients. To guarantee the quality of the educational activity, two patients per hour were scheduled, totalling eight consultations in 4 h. To participate in the process, specialists received a bonus of EU$ 30 or US$ 35 per work per hour; primary care physicians were given protected time for the joint consultations. As the confidence of the primary care teams to care for patients with asthma and COPD increased, joint consultations were replaced by round-table or case discussions, and consultations were only arranged for cases not resolved by these discussions. As required, contacts by phone or email between primary care teams and pulmonologists contributed to on-going support.

### Evaluation

The evaluation proceeded at two levels. The outcomes, reflecting the process of implementation, were as follows:

- Number of professionals trained at the workshops;
- Health professional knowledge of asthma and COPD, before and after training, using questionnaires (10 items multiple choice for asthma; 9 items and 1 subjective question for COPD) based on questions used to test undergraduate medical training. See Supplementary Appendices 1 and 2.
- Number of joint consultations and total number of patients seen.
- Number of round-table or case discussions
- Feedback from professionals
- The impact of the intervention was assessed by comparing routine data before and after the themed workshops, beginning in May 2013.

The primary health outcomes were as follows:

- The number of asthma and COPD referrals recorded by routine internal reports^17^ for the 2 years before and the 12 months after workshops.
- Number of beclomethasone propionate canisters dispensed (from pharmacy records;^17^ doctor prescribing data are not available) in the three territories for 5 months before the matrix-support workshops and compared with 5 months after.
- Asthma and COPD hospital emergency department visits for 5 months before and 7 months after the programme were collected from internal reports.^15^ These were only available for the ninth territory; data from the other two territories were not available for this study period.

### Data analysis

The data analysis is predominantly descriptive, except for the knowledge level and the referrals. The number of knowledge errors before and after training was compared using the non-parametric Mann–Whitney test. The analysis of the number of referrals compared accumulated over the 2 years before the date of the first seminar with cumulative referrals in the 12 months after the intervention. Results are expressed as the mean number of referrals per month to accommodate the different durations of the periods. Absolute values per period and referrals per month were compared in each territory using the *t*-test. Sigma Stat package software (Systat Software Inc., Richmond, CA, USA) was used. A *P* value<0.05 was considered significant.

## Funding

This project was made possible by an educational grant from the International Primary Care Respiratory Group (IPCRG) and an organisation-unrestricted grant from the Municipality of Sao Bernardo do Campo (Brazil).

## Figures and Tables

**Table 1:** Participation in the matrix-support intervention

*Territory/region*	*Physicians (*n*)*			*Nurses (*n*)*	*Others (*n*) (pharmacists, chest therapists)*
	*General physicians*	*Family physicians*	*Paediatricians*		
*Workshop attendees*					
3	02	13	02	15	10
7	06	17	04	20	11
9	02	7	03	12	8
Total	10	37	9	47	29
					
*Contributed to joint consultations*					
Total	7	37	1		
					
*Attended case discussions*					
	4	32	0		

**Table 2:** Content of the matrix-support workshops

Workshops (theoric)	Number	• 04 for each territory/region • Asthma (02) and COPD (02) repeated once
	Themed (8 h)	• CRD panorama and burden (local and national) • Role of PHC to prevent and control CRD • Overview of educational training program • CRD management • Practical case discussions • Regulatory protocol between levels of care
Presential BHU activities	Shared consultations (repeated one per physician) and round-table discussions (one or more per BHU);	

Abbreviations: BHU, basic health units; COPD, chronic obstructive pulmonary disease; CRD, chronic respiratory disease; PHC, primary healthcare.

## Notes

Supplementary Appendice 1

Supplementary Appendice 2
