# Supplementary material for: Implementation of ‘matrix support’ (collaborative care) to reduce asthma and COPD referrals and improve primary care management in Brazil: a pilot observational study
Source: NPJ Prim Care Respir Med. 2016 Aug 18;26:16047–. doi: 10.1038/npjpcrm.2016.47 (PMC4989903; doi:10.1038/npjpcrm.2016.47)
Supplement: Supplementary Appendice 1 [file npjpcrm201647-s1.doc]

Appendix 1- Asthma questionnaire

1. The main factor involved in the pathogenesis of asthma is?

1. Bronchospasm
2. Bronchial inflammation
3. Production of excessive bronchial mucus
4. Hypertrophy of glands of bronchial submucosal

2. Which statement below is correct?

1. Asthma is caused exclusively by hereditary factors
2. The household allergens have little role in the development of asthma and its symptoms
3. Smoking is an aggravating factor of inflammation and bronchial hyper-reactivity, with a consequent worsening of asthma symptoms;
4. The reduction in the prevalence of asthma in the last three decades is linked to environmental changes

3. In relation to asthma symptoms, the following statement is correct:

1. The main symptoms are: dyspnea, cough, wheezing and chest discomfort
2. Symptoms occur episodically ("crises") and appellant, more intense at night or in the early hours of the day
3. It is common to detect triggering or aggravating factors
4. All statements are correct

4 - The diagnosis of asthma is mainly clinical, though some additional tests may be useful. In relation to these tests, we can say:

1. Characteristically, the chest X-ray shows pulmonary hyper-inflation signals
2. Spirometry besides aiding in the diagnosis allows graduate the severity of bronchial obstruction
3. The measurement of peak expiratory flow (PEF) shows that asthma tends to PFE stability throughout the day
4. A normal spirometry exclude the possibility of asthma

5 - According to the GINA (Global Initiative for Asthma) one patient without treatment presenting daytime symptoms of asthma three times of week, should be classified of severity as:

1. Intermittent asthma
2. Mild persistent asthma
3. Moderate persistent asthma
4. Severe persistent asthma

6 - Regarding the maintenance treatment of asthma, is correct:

1. It aims to combat bronchial inflammation and therefore achieve disease control
2. Inhaled corticosteroids are the drugs of choice for maintenance treatment
3. Drug maintenance treatment is mandatory for all asthmatics
4. Affirmative A and B are correct

7. Select the correct statement:

1. The dosage of inhaled corticosteroids varies according to the type employed and mainly to the severity of the disease
2. There is no difference between topical potency of different inhaled corticosteroids
3. Inhaled corticosteroids are contraindicated in children
4. The most frequent adverse effect with the use of inhaled corticosteroids is related to suppression of the hypothalamic-pituitary-adrenal axis

8 - Patient asthmatic, despite regular use of beclomethasone (800 mcg / day), persists with frequent symptoms. What is the appropriate course of action in this case?

1. Increasing the dose of inhaled corticosteroids
2. Change inhaled corticosteroids for systemic corticosteroids
3. Add a beta two long-term agonist to inhaled corticosteroid
4. Add an inhibitor of leukotrienes to inhaled corticosteroid

9. What is the indicated therapy for asthmatic patient with mild symptoms 3 times / year?

1. Environmental control and short acting beta2 agonist as needed for symptoms relief
2. Environmental control and inhaled corticosteroids
3. Environmental control and theophylline
4. Environmental control and long acting beta2 agonist

10 – Regarding the management of asthma attacks, is correct:

1. Although systemic corticosteroids has immediate effect, the pulmonary function recovery time does not change when the drug is used early
2. The first step is bronchodilator administration, whose early start is a key to successful treatment
3. Supplemental oxygen is required for all patients
4. Aminophylline is a first-line drug for treating severe asthma attacks
