# Supplementary material for: Implementation of ‘matrix support’ (collaborative care) to reduce asthma and COPD referrals and improve primary care management in Brazil: a pilot observational study
Source: NPJ Prim Care Respir Med. 2016 Aug 18;26:16047–. doi: 10.1038/npjpcrm.2016.47 (PMC4989903; doi:10.1038/npjpcrm.2016.47)
Supplement: Supplementary Appendice 2 [file npjpcrm201647-s2.doc]

Appendix 2- COPD questionnaire

1 - The key points in the COPD definition includes:

1. systemic disease, completely irreversible, inflammatory, only smoking of origin;
2. pulmonary disease, partially reversible, inflammatory, only smoking of origin;
3. systemic disease, completely irreversible, inflammatory, predominantly smoking origin;
4. systemic disease, partially reversible, inflammatory, predominantly smoking origin.

2 - What is the epidemiological spirometry standard definition of COPD?

1. FEV1 / FVC <80% predicted and FEV1 <80% predicted
2. FEV1 / FVC <0.70 of predicted and FEV1 <80% predicted
3. FEV1 / FVC <0.70 pre-bronchodilator;
4. FEV1 / FVC <0.70 post-bronchodilator

3 - What are the scores of symptoms suggested by GOLD is?

1. MRC and CAT
2. MRC and Borg
3. Borg and CAT
4. Borg and St. George

4 - The GOLD functional classification of COPD severity is based on:

1. FEV1 / FVC ratio;
2. FEV1% predicted;
3. FVC% predicted;
4. FEV1 and FVC in spirometry figures

5 - What is considered stable COPD?

1. When patient only needs bronchodilators;
2. When patient is totally asymptomatic
3. When there is no indication to use any medication;
4. When patient presents stability of cough, phlegm and shortness of breath.

6 - Regarding smoking cessation in the treatment of patients with COPD:

1. It is the most effective (and cost-effective) single intervention to reduce exposure to risk factor
2. It is an expensive intervention and low cost-effectiveness
3. the dropout rate is very low, which makes it to be cost-effective
4. It is the most effective single intervention but with low cost-effectiveness to reduce exposure to risk factor

7 – Which outcome is common in frequent exacerbations COPD patients compared to those who did not exhibit:

1. worse quality of life
2. faster disease progression
3. increased mortality
4. all of the above are correct

 8 - Patients with COPD are at increased risk for the following co-morbidities, which should be actively sought, except:

1. diabetes
2. depression and anxiety
3. asthma
4. cardiovascular diseases

9. What are the indication parameters for long oxygen therapy?

1. PaO2 <7.3 kPa (65 mm Hg) or SaO2 <89% with or without hypercapnia
2. PaO2 <7.3 kPa (55 mm Hg) or SaO2 <88% without hypercapnia;
3. PaO2 <7.6 kPa (59 mm Hg) or SaO2 <87% with hypercapnia
4. PaO2 <7.3 kPa (55 mm Hg) or SaO2 <88% with or without hypercapnia

10 Do you feel secure to diagnose and treat COPD?

( ) Yes, why?

( ) No, why?
